# High Spatial Resolution Ion Imaging by Focused Electron-Beam Excitation with Nanometric Thin Sensor Substrate

**DOI:** 10.3390/s22031112

**Published:** 2022-02-01

**Authors:** Kiyohisa Nii, Wataru Inami, Yoshimasa Kawata

**Affiliations:** 1 Graduate School of Medical Photonics, Shizuoka University, 3-5-1 Johoku, Naka, Hamamatsu 432-8011, Japan; nii.kiyohisa.17@shizuoka.ac.jp; 2 Research Institute of Electronics, Shizuoka University, 3-5-1 Johoku, Naka, Hamamatsu 432-8011, Japan; inami.wataru@shizuoka.ac.jp

**Keywords:** light-addressable potentiometric sensor, high spatial resolution, electron-beam-induced current, thin sensor substrate, chemical imaging system, electron-beam addressable potentiometric sensor

## Abstract

We developed a high spatially-resolved ion-imaging system using focused electron beam excitation. In this system, we designed a nanometric thin sensor substrate to improve spatial resolution. The principle of pH measurement is similar to that of a light-addressable potentiometric sensor (LAPS), however, here the focused electron beam is used as an excitation carrier instead of light. A Nernstian-like pH response with a pH sensitivity of 53.83 mV/pH and linearity of 96.15% was obtained. The spatial resolution of the imaging system was evaluated by applying a photoresist to the sensing surface of the ion-sensor substrate. A spatial resolution of 216 nm was obtained. We achieved a substantially higher spatial resolution than that reported in the LAPS systems.

## 1. Introduction

An ion sensor is a device used to measure the concentration of a target ion. It is used in various applications, such as water quality surveys, blood chemistry, and the adjustment of a cell culture medium; pH is an important parameter in chemical measurement, and several ion sensors for detecting pH have been developed to date. In addition, miniature ion sensors, ion-sensitive field-effect transistors (ISFETs), have been developed, which further expand the application range of ion sensors [1,2].

Recently, ion sensors that can image the two-dimensional distribution of ions have been developed. Two-dimensional ion imaging provides visualization and dynamic analysis of processes such as electrolysis and corrosion [3]. It is also possible to obtain the distribution of ion concentrations in the vicinity of cells and measure the metabolic activity of living cells [4,5,6,7,8,9,10].

An ISFET array, which is a two-dimensional array of ISFETs, can be used for ion imaging. The ISFET array sensor achieves real-time ion imaging with a frame rate of 6100 fps [11]. CMOS ion-sensitive field-effect transistor (ISFET) arrays with column offset compensation have been proposed for long-term bacterial metabolism monitoring [12]. In addition, the graphene field-effect transistor arrays are proposed for real-time, high resolution, simultaneous measurement of multiple ionic species [13]. In ISFETs, the gate-insulating layer is directly immersed in the solution without using metal as the gate electrode of the metal oxide semiconductor field-effect transistor (MOSFET), and the ion concentration is measured from the change in drain current generated by the interface potential between the solution and the gate-insulating layer. The spatial resolution is limited by cell size, and can only be achieved at 9.22 μm × 7.56 μm with a chip size of 1024 × 1024 array [14].

A charge transfer-type ion-image sensor is capable of real-time, high-sensitivity measurement of ions. The concentration images of four kinds of ions (H^+^, K^+^, Na^+^, and Ca^2+^) could be obtained simultaneously through the CCD multi-ion-image sensor [15]. A proton image sensor was inserted into the brain to detect the changes of pH in the brain caused by any visual stimulation [16]. The pH value was converted into an electrical charge. The charges were then transferred to the output circuit for reading. The spatial resolution of a charge-transfer-type ion-image sensor was determined by the pixel size. A spatial resolution of 3.75 μm with 1.3 megapixels has been achieved in such a sensor [17].

In the LAPS, the ion concentration distribution can be imaged by scanning two-dimensionally with a laser beam. An analog micromirror was adopted for the raster scan of the sensor substrate, which enables high-resolution pH imaging with a frame rate of 8 fps [18]. The pH changes in the hippocampal formation of rats were obtained using an all-in-one pH probe [8]. The flat-band voltage shift due to the ions of the semiconductor in an EIS structure was measured with light. The spatial resolution of the LAPS depends on the spot size of the laser beam used for excitation, the thickness of the ion sensor substrate, and the diffusion of the carriers [19,20,21,22,23]. A spatial resolution of 0.8 μm was achieved by using the SOS substrate and the two-photon excitation method [24].

In this study, we developed an electron-beam-addressable potentiometric sensor (EAPS) to improve the spatial resolution of the ion sensor. In the EAPS, the light used in the LAPS is replaced by a focused electron beam [25,26]. The electron beam can realize a spot size of several nanometers. In addition, the silicon layer of the sensor substrate was thinned to suppress the diffusion of minority carriers. The pH measurement capability and spatial resolution were evaluated using an EAPS system.

## 2. Materials and Methods

### 2.1. Principle of Operation of an Electron-Beam-Addressable Potentiometric Sensor

#### 2.1.1. Method for Measuring the Distribution of the Ion Concentration

**Figure 1:**
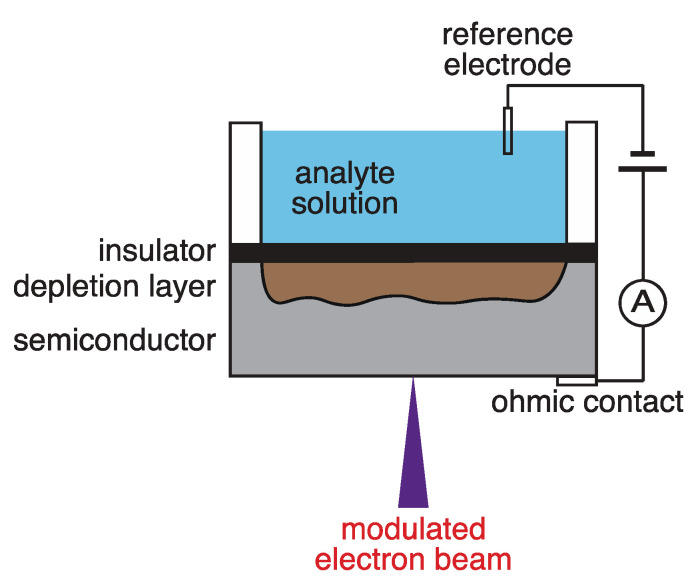
Schematic diagram of the electron-beam-excited ion-imaging system.

A schematic diagram of the electron-beam-excited ion-imaging system developed in this study is depicted in Figure 1. This system consists of an ion sensor substrate, analyte solution, reference electrode, power supply, ammeter, and electron beam for excitation. Insulating layers of SiO_2_ and Si_3_N_4_ were present on the surface of the sensor substrate. The Si_3_N_4_ layer in contact with the analyte solution acted as an ion-sensitive membrane. In addition, electrodes are attached to the rear surface of the sensor substrate through an ohmic contact. The Si_3_N_4_ on the surface of the ion sensor substrate forms a silanol group (Si-OH), and its state changes depending on the concentration of hydrogen ions in the analysis solution [27] as follows:

(1) $${\mathrm{SiOH}}_{2}^{+}↔\mathrm{SiOH}+H^{+},\;\mathrm{SiOH}↔{\mathrm{SiO}}^{-}+H^{+}$$

Consequently, the potential on the surface of the substrate changes according to the degree of ionic binding and dissociation. At the time of measurement, a power supply was used to create a depletion state by applying a voltage between the reference electrode and the electrode on the rear surface. When the potential on the substrate surface changes owing to the binding and dissociation of the ions, the thickness of the depletion layer formed at the substrate–insulating layer interface of the ion sensor substrate also changes. The thickness of the depletion layer was calculated from the amount of alternating current flowing through the circuit after irradiation using an electron beam. The thickness of the depletion layer depends on the ion concentration which can be calculated by measuring the thickness of the depletion layer.

#### 2.1.2. Measurement of the Depletion Layer Width with a Circuit Model and Electron Beam Irradiation

**Figure 2:**
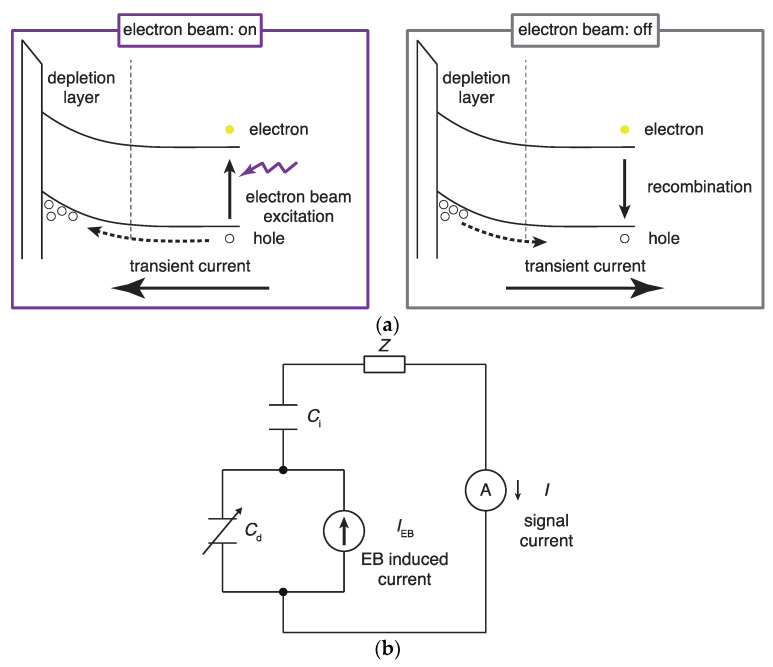
(**a**) Energy band diagrams explaining the generation of transient currents in an electron-beam-addressable potentiometric sensor after the electron beam is turned on and off. (**b**) Circuit model of an electron-beam-addressable potentiometric sensor.

A band diagram showing the alternating current generated by electron beam irradiation is presented in Figure 2a. When irradiated with an electron beam, electron-hole pairs are generated inside the semiconductor substrate that diffuses into the depletion layer. Electrons and holes are separated by an electric field in the depletion layer, resulting in a transient current. When the electron beam irradiation ceases, the excess holes in the depletion layer are removed by recombination. As a result, a transient current flows in the opposite direction [28,29].

The transient current generated through repeated irradiation by the modulated electron beam is represented by the alternating current (*I*_EB_) of the circuit model shown in Figure 2b. The alternating current (*I*_EB_) is divided according to the capacitances (*C*_d_) of the depletion layer and (*C*_i_) of the insulating layer in the area irradiated with the electron beam, and the alternating current flowing through the latter is measured externally as signal *I*. The series impedance *Z* includes the impedance of the solution, the resistance of the semiconductor, the contact resistance, and the input impedance of the transimpedance amplifier. If the series impedance *Z* is negligibly small, the alternating current *I* is ex-pressed using the following equation [28]:

(2) $$I=I_{\mathrm{EB}}\frac{C_{i}}{C_{i}+C_{d}}$$

#### 2.1.3. Relationship between the Ion Sensor Substrate and Spatial Resolution

The LAPS constitutes a system that uses light to excite electrons to measure the ion concentration. In the system developed in this study, a high spatial resolution is achieved by adapting the light used for excitation of the electron beam in the LAPS, such that it can form a smaller spot diameter. Spatial resolution is one of the most important indicators of the performance of chemical sensors. In the LAPS, the spatial resolution is determined by the range of diffusion of the charge carriers in the semiconductor layer if the light beam is sufficiently focused [20,22,30]. When irradiating an electron beam, the spatial resolution is defined according to the range of scattering of the electron beam and the diffusion of charge carriers. Under irradiation, the electron beam enters the silicon, generating electron-hole pairs that diffuse into the depletion layer. Even for a small spot diameter, a high spatial resolution cannot be achieved if the film is too thick. The spatial resolution can be improved by increasing the doping concentration of impurities or by reducing the thickness of the ion sensor substrate. It has been reported that a reduction in the thickness of the ion sensor substrate is a better method for improving the spatial resolution [19]. In our device, we prepared a window-structured ion sensor substrate by thinning the silicon-on-insulator (SOI) substrate through etching to achieve ion imaging with high spatial resolution.

### 2.2. Ion-Imaging System Based on Electron Beam Excitation

**Figure 3:**
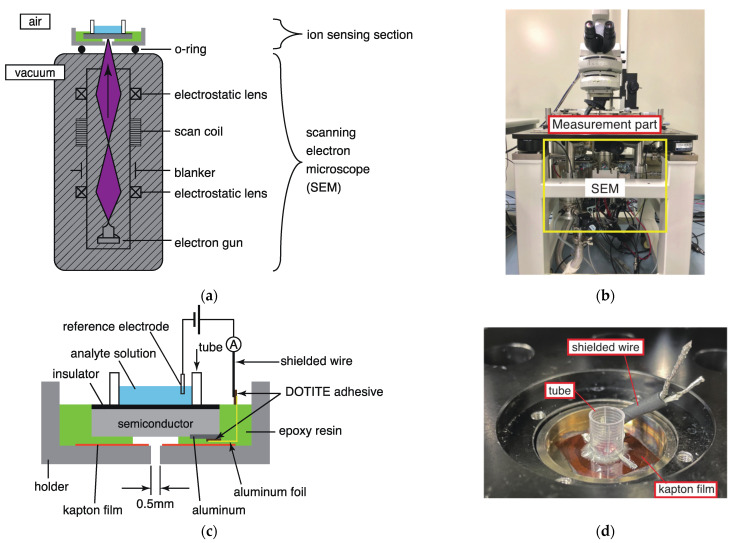
(**a**) Schematic diagram of the electron-beam-excited ion-imaging system, (**b**) image of the manufactured electron-beam-excited ion-imaging system, (**c**) enlarged view of the measuring section, and (**d**) image of the actual measuring section.

A schematic diagram of the ion-imaging system based on electron beam excitation is shown in Figure 3a. The electron beam emitted from the electron gun was converged by an electrostatic lens and irradiated on the ion sensor substrate. It is also possible to irradiate the sample while scanning the electron beam with a scan coil.

To irradiate the electron beam, it is necessary to install the rear surface of the ion sensor substrate under vacuum conditions. Therefore, as shown in Figure 3a, the inside of the electron microscope barrel is maintained under vacuum by installing an O-ring on the lower surface of the holder onto which the ion sensor substrate is attached. In the ion-sensing unit, only the rear surface of the ion sensor substrate was under vacuum. Therefore, even though the measurement system uses electron beam irradiation, it is possible to culture living cells on the surface of the ion sensor substrate and perform measurements in vivo.

The irradiation current and acceleration voltage were controlled by a control unit attached to an inverted scanning electron microscope (MINI-EOC, APCO Ltd., Tokyo, Japan). However, it was not possible to irradiate the electron beam while switching on and off with the control unit alone. Therefore, a function generator (AFG3021C, Tektronix, Beaverton, OR, USA) was connected to the blank part of the electron microscope (as shown in Figure 3a). By inputting an on/off electric signal, the electron beam can be irradiated while being modulated by the status of the signal.

An image of the manufactured electron-beam-excited ion-imaging system is shown in Figure 3b. The part shown in the yellow frame is the electron microscope, and the part shown in the red frame is the ion-measuring part. The measurement was performed by irradiating the ion sensor substrate fixed onto the holder with an electron beam emitted from an inverted scanning electron microscope.

An enlarged view of the measurement section is presented in Figure 3c. The aluminum electrode attached to the rear surface of the ion sensor substrate was glued with the conductive adhesive DOTITE (D-500, FUJIKURA KASEI Co., Ltd., Tokyo, Japan), and the electrode was extended using aluminum foil. The substrate with the extended electrodes was glued with epoxy resin (Araldite, Huntsman, The Woodlands, TX, USA) to a holder with a hole diameter of 0.5 mm for passing the electron beam. The Kapton film (DuPont de Nemours, Inc., Wilmington, DE, USA) was sandwiched between the substrate and holder to prevent electrical continuity between these components. The electrode was further extended from the aluminum foil with the help of the DOTITE adhesive using a shielded wire. An image of the actual measurement section is presented in Figure 3d. A cylinder intended to contain the analyte solution was attached to the epoxy resin. At the time of measurement, the analyte solution was placed in the cylinder, and a reference electrode was inserted. The measurement was performed by applying a voltage between the reference electrode and the electrode extending from the aluminum foil attached to the rear of the ion sensor substrate.

### 2.3. Fabrication of the Thin Ion Sensor Substrate

**Figure 4:**
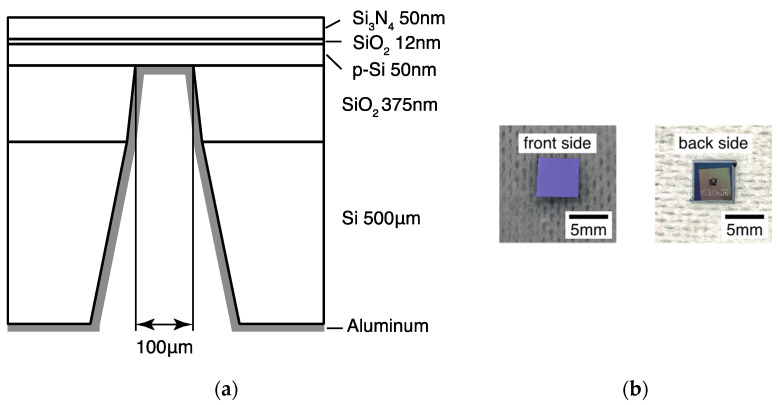
(**a**) Schematic diagram of the window-structure ion-sensor substrate configuration and (**b**) image of the front and rear surfaces of the manufactured substrate.

We formed a SiO_2_ and Si_3_N_4_ layer on an SOI substrate and fabricated a thin-film window-structured ion-sensor substrate on the rear surface by etching. A schematic diagram of the configuration of the window-structured ion-sensor substrate is shown in Figure 4a. A SiO_2_ layer of 12 nm was formed on the surface of a p-type SOI wafer (10–20 Ω·cm, Si = 50 nm) by thermal oxidation method, and a 50 nm Si_3_N_4_ layer was deposited via the low-pressure chemical vapor deposition (LP-CVD) method. The Si_3_N_4_ layer acted as an ion-sensitive membrane. The dimensions of the ion sensor substrate were 5 mm × 5 mm, and those of the window part were 100 μm × 100 μm. The prepared substrate was washed with piranha solution (H_2_SO_4_ (UN1830 H_2_SO_4_, FUJIFILM Wako Pure Chemical Corporation, Osaka, Japan): H_2_O_2_ (35% H_2_O_2_, Hirota chemical Industry, Co., Ltd., Tokyo, Japan) = 4:1) for 15 min. To form ohmic contacts on the rear surface of the substrate, the natural oxide film was removed by immersing it in a 1% HF (49% HF, Hirota chemical Industry, Co., Ltd., Tokyo, Japan) solution for 30 s. Subsequently, aluminum was attached as an electrode through vacuum vapor deposition. The thickness of the aluminum electrode was 30 nm. Images of the front and rear surfaces of the manufactured substrate are shown in Figure 4b. The front surface was flat, and the rear surface was dented because only the part used for imaging was thinned. Aluminum was deposited on the recessed window structure.

## 3. Results and Discussion

### 3.1. Measurement Results for Solutions with Various Values of pH

**Figure 5:**
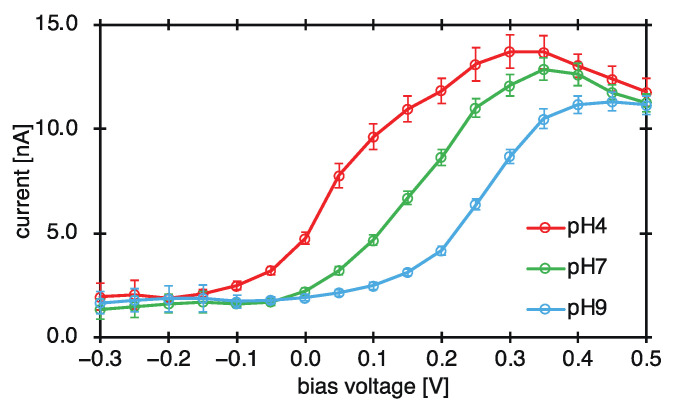
Bias voltage-current characteristics of the electron-beam-induced current.

The pH dependence of the bias voltage–current characteristics of the electron-beam-induced current was measured. The bias voltage–current characteristics acquired by the developed device are shown in Figure 5. The acceleration voltage of the electron beam was 5 keV, the irradiation current was 3.1 nA, and the modulation frequency was 820 Hz. pH standard solutions (pH standard solution, Horiba, Kyoto, Japan) with pH values of 4.01, 6.86, and 9.18 were used as the measurement solution. A water-based reference electrode (Ag/AgCl) (RE-1B, BAS, Tokyo, Japan) was used as the reference electrode. A dual-output power supply (E3620A, Keysight Technologies, CA, USA) was used as the power supply, and a current input preamplifier (LI-76, NF Corporation, Kanagawa, Japan) was used as the I-V amplifier.

**Figure 6:**
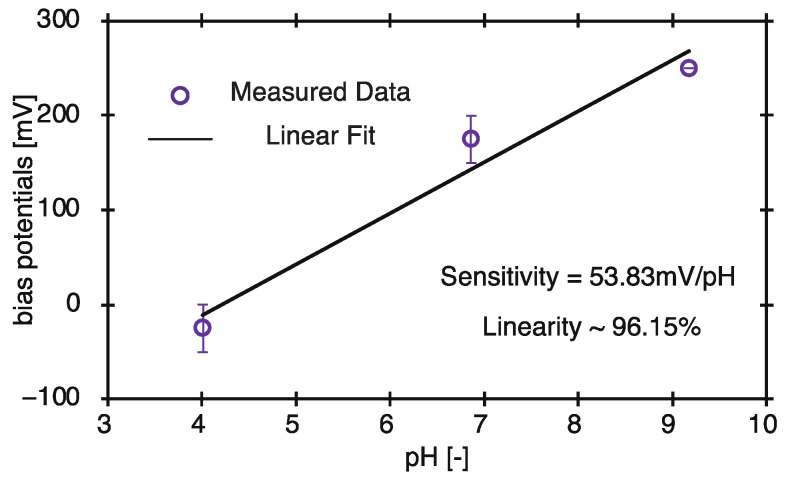
Sensitivity and linearity of the window-structure ion sensor.

At all three pH values, the bias voltage increased with a concomitant increase in the current through the circuit. This observation is in accordance with Equation (2). On applying a reverse bias voltage to the substrate, the depletion layer becomes thicker. This reduces the capacitance *C*_d_ of the depletion layer, and the capacitance of the insulating layer *C*_i_ and the alternating current *I*_EB_ are constant during measurement. Therefore, from Equation (2), the alternating current *I* measured by the external circuit increases. When a voltage of 0.1 V is applied, the resulting current flowing through the circuit differs for different values of pH. This is in accordance with Equation (1). The processes of ionic binding and dissociation, represented in Equation (1), depend on the hydrogen ion concentration of the standard pH solution. As a result, the potential on the surface of the substrate changed, and the thickness of the depletion layer also changed. When the voltage applied from the outside is constant, the AC current I measured by the external circuit also varies with the pH because the thickness of the depletion layer varies. This result clarifies how ion concentration can be measured. The bias voltage corresponding to the inflection point in each of the curves in Figure 5 was calculated to calculate the pH sensitivity and linearity of the prepared ion-sensor substrate. The relationship between the bias voltage and pH, as represented by the inflection points obtained by calculation, is shown in Figure 6.

The resulting pH sensitivity and linearity were calculated to be 53.83 mV/pH and 96.15%, respectively. These values are in good agreement with the ideal Nernstian values (59.16 mV/pH at 25 °C).

### 3.2. Evaluation of the Spatial Resolution

**Figure 7:**
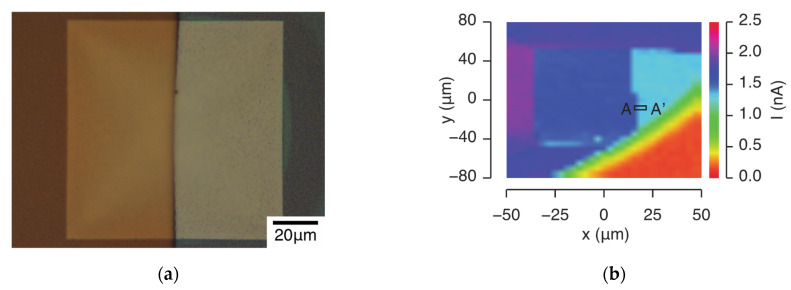

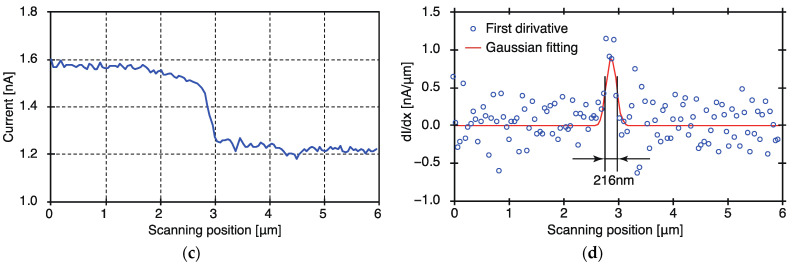
(**a**) Optical microscope image of the ion sensor substrate after photolithography. The photoresist is applied to the left half. (**b**) Electron-beam-induced current image, (**c**) intensity line-profile, and (**d**) first derivative of the curve shown in (**c**).

To evaluate the spatial resolution, an ion sensor substrate with half the surface covered with a photoresist was manufactured through a photolithography process [31,32,33]. The manufacturing procedure included the following steps: (1) coating a photoresist layer, (2) ultraviolet exposure, and (3) developing the image. A positive photoresist, OFPR800 (Tokyo Ohka Kogyo Co., Ltd., Kanagawa, Japan), was formed via spin coating using a spin coater (ACT-220DII, Active, Saitama, Japan). The film was formed at a rotation speed of 2600 rpm with a formation time of 16 s. Subsequently, using a manual mask aligner (MJB4, SUSS MicroTec, Garching, Germany), irradiation with ultraviolet rays for 3 s ensured that the resist remained on half of the window part of the substrate. The substrate, which was exposed to ultraviolet rays, was immersed in a developing solution (NMD-3, Tokyo Ohka Kogyo Co., Ltd., Kanagawa, Japan) for 40 s. After development, it was rinsed by immersion in pure water for 30 s. After development, the photoresist was baked and hardened by baking it for 300 s on a hot plate heated to 135 °C. After baking, the knife-edge was confirmed using a semiconductor/flat-panel-display (FPD) inspection microscope (MX51, OLYMPUS, Tokyo, Japan). An image of the ion-sensor substrate after photolithography is shown in Figure 7a. An edge exists at the center of the sensor. The left side was covered with a photoresist. The Si_3_N_4_ layer was exposed to the right side. The thickness of the photoresist at the knife-edge was measured as 1.35 μm using a profilometer (Alpha-step IQ, KLA-Tencor, Milpitas, CA, USA).

An electron-beam-induced current image obtained using a photoresist-coated ion-sensor substrate is presented in Figure 7b. The electron-beam-induced current image was obtained from the current flowing through the circuit by scanning the substrate two-dimensionally with an electron beam. The acceleration voltage of the electron beam was 5 keV, the irradiation current was 1.0 nA, and the modulation frequency was 1 kHz. A standard pH solution (pH 6.86) was used as the measurement solution. The bias voltage was set at 0 V. The number of pixels was 32 × 32 and the dwell time was 0.02 s. The dark blue part of the figure corresponds to the photoresist-coated part, and the light blue part corresponds to the non-photoresist-coated part. The red part on the lower right corresponds to the holder attached to the ion-sensor substrate. The electron-beam-induced current image clearly shows that the current in the part where the photoresist is applied differs from that in the part where the photoresist is not applied. In addition, an electron-beam-induced current image was acquired in the region of the black rectangle A-A’ in Figure 7b, corresponding to the edge part. The number of pixels was 128 × 1. The intensity line profile of the acquired electron-beam-induced current image is shown in Figure 7c. The spatial resolution was determined from the FWHM of the first derivative of the intensity line profile. This method produced a spatial resolution of 216 nm, as indicated by the vertical lines in Figure 7d.

To estimate the extent of scattering of the electron beam irradiated onto the ion sensor, the scattering state of the electron beam at the thickness of the substrate used was calculated using the Monte Carlo simulation method. The relationship with the FWHM was investigated by calculating the scattering range of the electron beam that reached the depletion layer formed at the interface between the Si and SiO_2_ layers. The free software package CASINO (Monte Carlo simulation of electroN trajectory in sOlids) was used for the simulation [34].

**Figure 8:**
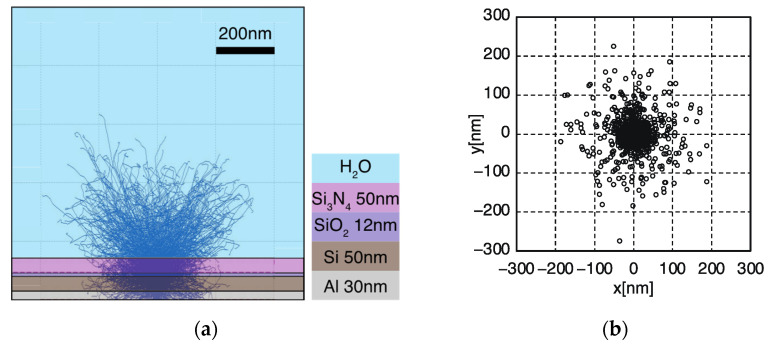
(**a**) Monte Carlo simulation results of electron beam scattering on the ion-sensor substrate and (**b**) electron beam passage position at the Si/SiO_2_ interface.

The results of the electron beam scattering simulations are shown in Figure 8a. The composition of the ion-sensor substrate was Si_3_N_4_(50 nm)/SiO_2_(12 nm)/Si(50 nm)/Al(30 nm) and water (H_2_O) was placed on the sample side. The electron beam irradiation conditions were calculated under the assumption that the acceleration voltage was 5 keV, the number of calculated electrons was 1000, and the diameter of the electron beam was 20 nm.

The spread of the electron beams in the depletion layer was calculated from the Monte Carlo simulation results of the electron beam scattering. The electron beam passage position at the Si/SiO_2_ interface is shown in Figure 8b. The origin was located at the position of the electron beam irradiation. When an electron beam with an acceleration voltage of 5 keV was irradiated, the scattering range of the electron beam at the Si/SiO_2_ interface became 276 nm at the maximum displacement from the origin. The FWHM was calculated from the result of the electron beam scattering simulation in the same way as the spatial resolution evaluation by the first-derivative FWHM method in the experiment. The FWHM was found to be 64 nm. This value was given only by electron scattering for the excitation of carriers, and did not include the diffusion and drift effect of the excited carriers.

In the system developed in this study, electron-hole pairs were generated along with the scattering of electron beams. These charged particles diffused into the depletion layer. Consequently, the spatial resolution was negatively impacted. In addition, carriers trapped on the semiconductor surface by the electric field of the depletion layer can diffuse parallel to the surface [19]. It is considered that such diffusion caused the carriers to spread laterally; the spatial resolution obtained in the experiment was lower than the FWHM obtained in the Monte Carlo simulation.

From the simulation results depicted in Figure 8a, it can be seen that the scattered electron beams reached the water region. If imaging is performed under these conditions, the cells are damaged by the electron beam. A higher spatial resolution may be achieved by suppressing the scattering range of electron beams. Therefore, it is necessary to perform imaging under conditions that do not damage the cells, while suppressing the scattering range of electron beams by lowering the acceleration voltage.

## 4. Conclusions

A window-structure ion-sensor substrate was prepared with the aim of increasing the spatial resolution of the electron-beam-addressable potentiometric sensor. The pH values of the solutions were measured using the substrate. A Nernstian-like pH response with a pH sensitivity of 53.83 mV/pH and linearity of 96.15% was obtained. In addition, the spatial resolution was evaluated by applying a photoresist to the sensing surface of the ion-sensor substrate. The spatial resolution was 216 nm. From the scattering range of the electron beam by Monte Carlo simulation, the FWHM was 64 nm. In this system, electron-hole pairs were generated along with the scattering of electron beams, and these pairs diffused into the depletion layer, negatively impacting the spatial resolution. In addition, carriers trapped on the semiconductor surface owing to the electric field of the depletion layer could diffuse parallel to the surface. It is considered that such diffusion caused the carriers to spread laterally; consequently, the spatial resolution obtained in the experiment was lower than the FWHM obtained in the Monte Carlo simulation. A high spatial resolution was achieved by thinning the substrate.

As reported for LAPS systems, it is considered that higher spatial resolution could also be achieved in our system by suppressing the diffusion in the lateral direction by increasing the doping concentration of impurities. However, most minority carriers generated by excitation are lost by recombination with the majority carriers in the background, causing a decrease in the signal-to-noise ratio. Thus, a higher spatial resolution is achieved at the expense of the signal-to-noise ratio. Therefore, it is necessary to adjust the doping concentration of the impurities by considering the signal-to-noise ratio.

To achieve a high spatial resolution, it is necessary to increase the doping concentration of impurities and to use higher acceleration voltage to focus the electron beam. However, when an electron beam is irradiated with a high acceleration voltage to sensor substrate, the electron beam reaches the insulating layers SiO_2_ and Si_3_N_4_, and the sensor chip is charged. The charging of the sensor chip affects the pH measurement. Therefore, it is necessary to investigate the effect of the charging on the substrate to the pH measurement. When the film thickness is fixed, damage to the sample can be eliminated, and the loss of spatial resolution can be suppressed by using an acceleration voltage that does not reach the SiO_2_ layer. In the future, we aim to further improve the spatial resolution of our sensor by using an acceleration voltage that does not reach the SiO_2_ layer.

## Data Availability

Not applicable.
